# Pattern of Detections Across Multiple Environmental Messenger RNAs (e‐mRNAs) in Stressor‐Exposed Zebrafish (*Danio rerio*)

**DOI:** 10.1002/ece3.72986

**Published:** 2026-01-23

**Authors:** Denise L. Lindsay, J. Erik Mylroie, Kurt A. Gust, Elijah M. Cowan, Richard F. Lance

**Affiliations:** ^1^ Environmental Laboratory United States Army Corps of Engineers Research and Development Center Vicksburg Mississippi USA; ^2^ Department of Biology Millsaps College Jackson Mississippi USA; ^3^ Sinnhuber Aquatic Research Laboratory, Department of Environmental and Molecular Toxicology Oregon State University Corvallis Oregon USA

**Keywords:** biomonitoring, environmental messenger RNA (e‐mRNA), environmental RNA (eRNA), gene‐specific e‐mRNA degradation, perfluorooctanesulfonic acid (PFOS), peroxisome proliferator‐activated receptor (PPAR) pathway

## Abstract

Environmental RNA (eRNA) is gaining ground as an environmental monitoring tool. Whereas eDNA is mainly utilized for species detection, eRNA may provide additional classes of inference. The comparatively more rapid signal decay rates of eRNA provide narrower temporal windows for species presence, while detection of environmental messenger RNAs (e‐mRNAs) could provide evidence of genomic responses to environmental stressors. We explored e‐mRNA as an environmental tracer for stress imposed on animal populations by investigating the decay dynamics of e‐mRNA gene detections from target organism presence to recent presence. We tested seven select e‐mRNAs of known molecular targets of perfluorooctanesulfonic acid (PFOS) toxicity in tanks containing zebrafish ( *Danio rerio* ) exposed to an environmentally relevant concentration of PFOS. eRNA samples were collected just prior to fish removal following a 21‐day exposure and continued over nine timepoints across 3 days. The quantity and quality of total eRNA declined over time for both treatments, but were still detectable at 72 h post fish removal. The PFOS exposure failed to elicit observable shifts in e‐mRNA target concentrations compared to control tanks, perhaps because the selected gene targets are primarily responsive to PFOS in liver and kidney, which may not contribute strong eRNA signatures. Detection rates for all e‐mRNAs dropped significantly beyond 3 h post fish removal, with most being undetectable by 72 h. The signal lifespan of e‐mRNAs in this study implies that the detection of such traces will be a strong indicator of target organism presence (or recent presence), and that given the right combination of stressor concentrations, impacted tissues or organs, and gene targets, contaminant impacts on organism health should be detectable in environmental samples. Future studies targeting toxicologically effective stressor doses for well‐established gene targets will be an important advancement in establishing the utility of e‐mRNA as a noninvasive environmental stressor monitoring tool.

## Graphical abstract

We evaluated e‐mRNAs of known molecular targets of perfluorooctanesulfonic acid (PFOS) toxicity in zebrafish in a controlled laboratory experiment. The detection patterns of e‐mRNAs in our study imply that diminishing detection of these traces over time is a strong indicator of target organism presence (or very recent presence), providing a narrower timeframe of detection than eDNA provides. Our study also suggests that contaminant impacts on organism health should be detectable in water samples, given the right combination of stressor concentrations, impacted tissues or organs, and gene targets.

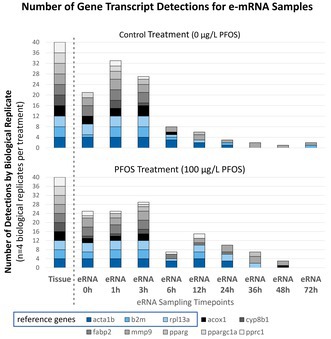

## 1 Introduction

The development of environmental RNA (eRNA) as a novel tool for environmental monitoring—one that provides more informative environmental characterizations than those currently obtained through studies of environmental DNA (eDNA) – is well underway. Before recent eRNA studies, RNA (especially messenger RNA; mRNA) was expected to degrade more rapidly than DNA and perhaps not be detectable as eRNA in water samples. An initial concern surrounding the use of eRNA for organismal surveys, particularly for studies of macroscopic eukaryotes that would not be captured intact in environmental samples, was that eRNAs would decline to undetectable concentrations too rapidly to be of much use (Cristescu 2019; Sachs 1993; Peltz et al. 1991).

However, recently published information has demonstrated that this is not the case. In the past few years, studies of eRNA have moved from investigations of simple, highly controlled, exploratory and/or methodological laboratory experiments (Hiki and Jo 2025; Xu and Asakawa 2025; Lindsay et al. 2024; Jo et al. 2023; Giroux et al. 2022; Kagzi et al. 2022; Marshall et al. 2021; Tsuri et al. 2021; Yates et al. 2021; Wood et al. 2020; Zaiko et al. 2020; Cristescu 2019; von Ammon et al. 2019; Pochon et al. 2017) to more complex investigations able to discriminate metabolically active, living organisms (Littlefair et al. 2022; Miyata et al. 2021), organismal life stages (Parsley and Goldberg 2024), and changes in gene expression (Hechler et al. 2022; Stevens and Parsley 2023).

Because RNAs are more closely associated with metabolically active cells, eRNA detections are expected to be associated with only very recently alive (and recently present) organisms (Littlefair et al. 2022; Cristescu 2019; Pochon et al. 2017). Lindsay et al. 2024 and Marshall et al. 2021 have demonstrated that eRNA of target organisms can be detected up to five days after removal of the organism; Tsuri et al. (2021) detected a variety of target organism‐specific messenger eRNAs (e‐mRNAs), and Miyata et al. (2021), Parsley and Goldberg (2024), and Hechler et al. (2022) have used e‐mRNAs to investigate a variety of organism‐specific gene responses. These recent studies have shown that eRNA detections of target organisms are possible and might provide higher confidence of living organismal presence than is inferable from eDNA (Lindsay et al. 2024; Littlefair et al. 2022; Cristescu 2019; Pochon et al. 2017), along with the addition of unique information on organismal state discernible by changes in gene expression.

Changes in gene expression related to a stressor indicate the adverse biological response of an organism exposed to a substance or circumstance (and not specifically an increase in cortisol). The possibility of assessing changes in environmental conditions based on the detection of stressor‐associated mRNA in simple water samples would be extremely useful to rapidly and efficiently detect the presence and impacts of stressors in natural environments, providing a novel biomonitoring tool. Studies such as this one are critical to understanding whether eRNA fulfills the potential of being a new type of environmental tracer, providing novel information relative to eDNA, and either surpassing or substantially complementing traditional environmental toxicology field monitoring methodologies. The ability to monitor changes in eRNA for genes with known responses to contaminants, pathogens, or invasive species would provide a means for the early detection of environmental stressors. As discussed in Tsuri et al. (2021) and Yates et al. (2021), because there are many forms of RNA that have a much richer suite of functions than DNA (e.g., protein coding, ribosomal scaffolding, gene regulation), eRNAs may directly provide a wealth of information about organismal states and activities, and indirectly offer information about associated factors (e.g., climate, pollution, and competition). Further, the rapid degradation of RNA in the environment provides opportunities to pinpoint a temporal window for environmental stressor exposures where eRNA signatures represent “current events” in the environment and not the long‐term legacy of exposures. Ultimately, strategic identification of eRNA with known functional responses to contaminants of concern can enable environmental monitoring that seamlessly integrates contaminant identification and biological impacts to advance site‐specific risk characterization.

Because inducible stress responses in zebrafish ( *Danio rerio* ) (F. Hamilton 1822) have been revealed through the differential mRNA expression of several genes (Mylroie et al. 2021; Gust et al. 2017) and because *D. rerio* ‐specific RNA has previously been detected in water samples (Lindsay et al. 2024; Wood et al. 2020), we expect that RNA associated with stress response in zebrafish can be detected in environmental samples using reverse transcriptase quantitative real‐time PCR (RT‐qPCR) assays for specific genes. Our pilot study (Lindsay et al. 2024) explored the detection of zebrafish eRNA in controlled laboratory experiments, tracking the degradation of several eRNA classes over time, and compared decay rates of synthetic eRNA and eDNA under various environmental stressor conditions. We found that the comparative decay rates among eRNA classes and sequences (i.e., loci) were consistent across degradation factor treatments (temperature and bacterial loads), indicating a significant role for RNA sequence in determining degradation dynamics—while structural eRNA was detectable up to 96 h, e‐mRNA was largely undetectable after 48 h. Based on prior eRNA degradation studies (Xu and Asakawa 2025; Lindsay et al. 2024; Scriver et al. 2023; Giroux et al. 2022), eDNA degradation studies (Strickler et al. 2015; Barnes et al. 2014; Lance and Guan 2020; Lance et al. 2017), RNA degradation studies (Houseley and Tollervey 2009; Sachs 1993), and mRNA degradation studies (Gallego Romero et al. 2014; Opitz et al. 2010), we expected to see variations in eRNA decay rates resulting from factors such as nucleotide sequence, sequence length, secondary, and tertiary structures, which may affect RNA stability in an aquatic matrix. The class of RNAs being studied here, e‐mRNA, is expected to decline rapidly with time, with increased variability in copy number concentrations and detection over time, and only samples collected within 48 h of fish removal will provide gene detection. We sought to address a practical question related to e‐mRNA as an evidentiary tracer for stress imposed on animal populations by investigating the decay dynamics and patterns in relative e‐mRNA detections and concentrations after organisms are no longer present. We hypothesized that *D. rerio* ‐specific e‐mRNA would exhibit varied decay rates and, hence, detectability over time, by gene. Our study contributes to the growing body of knowledge about macroscopic eukaryotic eRNA by determining whether stressor effects on target organisms can be detected over varying lengths of time since removal of the target organism from the environment.

## 2 Materials and Methods

### 2.1 Zebrafish Aquaculture and Breeding

Experimentation was conducted in accordance with the US National Research Council's Guide for the Care and Use of Laboratory Animals (Institute of Laboratory Animal Resources (US) 1986), the US Public Health Service's Policy on Humane Care and Use of Laboratory Animals (National Institutes of Health (US) 1986), and the Guide for the Care and Use of Laboratory Animals under the US Army Engineer Research and Development Center *Institutional Animal Care and Use Committee (IACUC) Protocol* #EL‐6008‐2022‐1.

Wild‐type AB strain adult zebrafish (Zebrafish International Research Center, Eugene, OR, USA) were housed in 2.8 L polycarbonate tanks (Tecniplast, West Chester, PA, USA) filled (to 2.5 L) with reverse osmosis water (RO water) supplemented with Instant Ocean salts (IO) and sodium bicarbonate. Tanks were housed in a chamber with a 14:10 h light: dark cycle, and the target water quality parameters of conductivity of 750 μS/cm, pH of 7.5, and temperature of 27.5°C. Individual tanks were aerated to ensure proper dissolved oxygen content throughout the exposure. Adult fish were fed GEMMA 500 μm fish food (Skretting, Tooele, UT, USA) twice daily on weekdays and once daily on weekends. Tanks were cleaned twice daily, followed by an ≈80% solution exchange on weekdays and once daily on the weekends. Fish were not exchanged during the exposure (all fish remained in assigned replicates/tanks), but tanks were rotated on weekends (used tanks were fully cleaned after fish were moved to clean tanks).

Fish used for this exposure were generated by breeding adult fish in an iSpawn (Tecniplast) breeding chamber according to the manufacturer's recommendations (2:1 ratio of males: females, separating fish the day before spawning with female fish above, and spawning during the dawn light cycle) for 30 min. Embryos were then collected and surface sterilized following Varga and Murray (2016). Zebrafish embryos were reared for 4 months (until they reached sexual dimorphism), then bred to confirm sexual maturity before beginning the PFOS exposure experiment.

### 2.2 Analytical PFOS Chemistry

The heptadecafluorooctanesulfonic acid potassium salt (PFOS; > 98% purity; CAS no. 2795‐39‐3, Product 77282, Lot BCBS9941) used in this experiment was obtained from Sigma‐Aldrich (Saint Louis, MO, USA). A stock solution of 100 mg/L of PFOS was prepared in ultra‐pure water (Milli‐Q purification system, MilliporeSigma, Burlington, MA), and the stock solution was analyzed in triplicate via liquid chromatography–triple quadrupole tandem mass spectrometry to obtain a measured concentration for use in subsequent PFOS exposure solution preparation. The 100 μg/L PFOS solutions used for this exposure were made in 20 L bulk batches by diluting the 100 mg/L PFOS stock in IO‐supplemented RO water (conductivity≈750 μS/cm and pH≈7.5), while the control treatment included only IO‐supplemented RO water with identical water quality targets.

Water samples for quantifying PFOS levels in tank waters (*n* = 5 samples per sampling event) were collected on Days 0, 1, 11, and 21 of the exposure. Water samples for Day 0 were obtained from the bulk solution used to fill the tanks, but all other water samples were obtained from tank replicates. Whole fish (*n* = 16, one male and one female from each tank) were collected at the conclusion of the exposure to confirm accumulation of 100 μg/L PFOS in fish tissues. Chemical analyses (*see* Gust et al. 2024 for methods) were completed for the water and tissue samples by the US Army Engineer Research and Development Center Environmental Laboratory Environmental Chemistry Branch.

### 2.3 PFOS Exposure

The PFOS exposure experiment was conducted in a controlled laboratory environment to limit unaccounted variance in conditions and fish stressors. Zebrafish in four replicated PFOS (100 μg/L PFOS) and four replicated control (0 μg/L PFOS) exposures, each holding 24 fish (12 male and 12 female) in 8 L tanks, were maintained for 21 days. Water exchanges ceased 2 days before the end of the exposure to allow for greater accumulation of eRNA in tank waters. Aeration ceased when zebrafish were removed from the tanks at the end of the exposure. Water sampling commenced immediately before zebrafish removal (timepoint ‘eRNA 0 h’) and at 1, 3, 6, 12, 24, 36, 48, and 72 h post fish removal (timepoints ‘eRNA 1–72h’). Water samples consisted of triplicate 50 mL volumes, representing technical replicates within the four biological replicate tanks per treatment. Water collections were conducted using sterile, disposable 50 mL pipettes and stored in 50 mL sterile conical tubes at −80°C until extraction. Prior to sampling each tank, water was gently stirred using the pipette to resuspend sloughed cells and any other eRNA‐bearing materials, without scraping the bottom or edges of the tank (so as not to disrupt any biofilm present). All fish were sacrificed at the conclusion of the exposure, and measurements (weight and length) were taken for each individual. Average weight and length of the exposed zebrafish were analyzed by treatment, with unpaired *T*‐tests conducted to compare treatment effects.

### 2.4 | Zebrafish Tissue and eRNA Extraction

Samples were processed in batches by treatment (control vs. PFOS‐exposed) and the nine sampling timepoints (eRNA 0 h—eRNA 72 h) to prevent cross‐contamination during RNA extraction and cDNA synthesis. Protocols for eRNA extraction, cDNA synthesis, and qPCR were initially optimized (thaw process, incubation times, and template amounts) using non‐study water samples from zebrafish holding tanks. Whole fish were collected from both treatments (*n* = 2 per treatment) at the conclusion of the exposure for use as tissue positive controls in RT‐qPCR (e.g., to ensure non‐detections were real and not due to failed RT‐qPCR) and for comparison to e‐mRNA detections.

The eRNA extraction began with the thawing of sample tubes in a cold water bath for 1 h, followed by centrifugation at 5000 *g* and 10°C for 10 min to create a pellet. The supernatant was carefully discarded, and the pellet was processed with the Quick Miniprep Plus Kit (ZYMO Research, Irvine, CA, USA) for RNA/DNA co‐extraction using the Cells protocol with modification of incubation times to maximize template RNA quantity and quality. After an initial 15 min air dry of the pellet, it was resuspended in 300 μL DNA/RNA Lysis Buffer and incubated at room temperature for 30 min. All other steps followed the published protocol. Genomic DNA elimination steps were performed twice on the RNA samples: first during the ZYMO extraction (optional DNaseItreatment) and again during cDNA synthesis. cDNA synthesis was completed using the RT^2^ First Strand Kit (QIAGEN) and utilized the maximum input of template (8 μL) for each eRNA sample due to low RNA concentrations, which was not unexpected given the nature of eRNA. Tissue RNA extractions (using white muscle tissue from the tail section of the collected whole fish) also used the ZYMO Kit, but with the Tough‐to‐Lyse Samples protocol and no modifications, other than the addition of the genomic DNA elimination steps as performed for the eRNA samples.

### 2.5 RNA Quantification

RNA concentration (ng/μL) and quality (260/280 ratio) were determined using a NanoDrop 2000 spectrophotometer (ThermoScientific, Waltham, MA, USA) for all samples. To determine RNA Integrity Number (RIN) scores, eRNA samples were run on a 2200 TapeStation High Sensitivity ScreenTape System (Agilent Technologies, Santa Clara, CA, USA) following manufacturer protocols.

### 2.6 Selection of Zebrafish Functional and Reference Genes for Investigation

The target genes selected for this study were associated with the peroxisome proliferator‐activated receptor (PPAR) pathway—regulators of lipid metabolism and energy release through lipid breakdown, as well as lipid storage and adipogenesis—though PFOS indiscriminately affects many different biological functions and pathways (“shotgun effect”). Thirteen custom QIAGEN *Danio rerio* RT^2^qPCR Primer Assays were selected such that one gene from each PPAR function/pathway was represented from the 39 genes found to be differentially expressed in a previous study of zebrafish embryos exposed to PFOS (Table 1; Mylroie et al. 2021). This preliminary set of genes was assayed to explore their applicability to our e‐mRNA study using tissue samples from a multigenerational PFOS study (Gust et al. 2024) and a set of non‐experimental eRNA samples (water samples collected from zebrafish holding tanks) to determine which genes would amplify eRNA, providing a background proof‐of‐concept before running our study samples (Table 1). In addition to the seven PPAR pathway genes, e‐mRNAs from three reference genes were assayed in this study as internal standards (Table 2), which were confirmed to have stable expression across all experimental conditions.

**TABLE 1:** Initial testing of 13 PPAR pathway genes with non‐experimental *Danio rerio* tissue and e‐mRNA samples to determine the likelihood of amplification in experimental e‐mRNA samples.

Sample	*acox1*	*cyp8b1*	*fabp2*	*mmp9*	*pparg*	*ppargc1a*	*pprc1*	*apq7*	*cd36*	*hspd1*	*mlycd*	*ppya*	*ucp1*
PFOS Tissue 1	+	+	+	+	+	+	+			+	+	+	+
PFOS Tissue 2	+	+	+	+	+	+	+	+	+	+	+	+	
Control Tissue 1	+	+	+	+	+	+	+		+	+	+		
Control Tissue 2	+	+	+	+	+	+	+		+	+	+		+
Control e‐mrna 1	+		+		+	+							
Control e‐mrna 2	+		+	+		+	+						
Control e‐mrna 3	+	+	+	+	+		+						
Control e‐mrna 4	+	+	+	+	+		+						
Control e‐mrna 5	+	+	+	+	+		+						+

*Note:* Genes not selected for use in experimental study samples are highlighted in gray. Tissue samples collected from zebrafish exposed to PFOS at 0 μg/L (control) and 100 μg/L (PFOS‐exposed) originated from a study by Mylroie et al. (2021). Control e‐mRNA water samples were collected from *D. rerio* holding tanks (0 μg/L PFOS). Positive detection (one or more amplifications in three technical replicates) in RT‐qPCR assays is noted with a “+”.

**TABLE 2:** Gene targets selected for *Danio rerio* e‐mRNA investigations. The “stressor related genes” represent genes involved in the peroxisome proliferator‐activated receptor (PPAR) signaling pathway, a principal molecular pathway involved in PFOS‐related toxicological effects, and the “reference genes” represent genes with constitutive expression in zebrafish. Gene symbols, descriptions, biological functions, origin (tissues in which mRNA is expected to be actively expressed), and likelihood of detection in water samples/environmental RNA are provided for context.

Gene symbol	Description	Biological function	Origin	Likelihood of eRNA detection
**Stressor‐related genes**				
*acox1*	PAX interacting protein 1 (with transcription‐activation domain)	Rate‐limiting enzyme in peroxisomal beta‐oxidation of very long‐chain fatty acids	Highly expressed in liver; glandular cells of the small intestine and duodenum	Likely detectable in feces
*cyp8b1*	Cytochrome P450, family 8, subfamily B, member 1	Catalyzes reactions involved in drug metabolism and synthesis of cholesterol, steroids, other lipids; bile acid biosynthesis pathway	Endoplasmic reticulum of liver; hepatocytes	Possibly detectable in feces or urine
*fabp2*	Fatty acid binding protein 2, intestinal	Moves lipids from the intestinal lumen to enterocytes and binds superfluous fatty acids to maintain steady collection in epithelium	Expressed in proximal enterocytes of duodenum and small intestine	Likely detectable in feces
*mmp9*	Matrix metalloproteinase 9	Activates the vascular endothelial growth factor protein family and thereby promotes angiogenesis in tumors; involved in the degradation of the extracellular matrix	Found most prominently in immune cells and tissues; digestive tract tissues: stomach, duodenum, small intestine, colon, cloaca; secreted by neutrophils, macrophages, fibroblasts	Likely detectable in feces
*pparg*	Peroxisome proliferator‐activated receptor gamma	Nuclear hormone receptor that regulates fatty acid storage, glucose metabolism, and gene expression; promotes glycolysis; adipogenesis/lipid metabolism, glucose metabolism/insulin signaling pathways, immune response, cell differentiation/apoptosis/tumor suppression	Adipose tissue, colon, macrophages; also found in high levels in cells associated with the female reproductive system (trophoblasts)	Likely detectable in feces and female reproductive cells
*ppargc1a*	Peroxisome proliferator‐activated receptor gamma, coactivator 1 alpha	Regulates cellular energy metabolism, increases transcriptional activity of *pparg* and thyroid hormone (strongly induced by cold exposure and thus adaptive thermogenesis); mitochondrial biogenesis	Highly expressed in the liver and in tissues with high energy demands: heart muscle, skeletal muscle, etc.; highly expressed in the parathyroid gland	Possibly detectable in feces or urine
*pprc1*	Peroxisome proliferator‐activated receptor gamma, coactivator‐related 1	Coactivator during transcriptional activation of nuclear genes related to mitochondrial biogenesis and cell growth	Highly expressed in mitochondria‐enriched tissues with high energy demands, such as brown adipose tissue, heart, and slow‐twitch skeletal muscle	Possibly detectable in urine
**Reference genes**				
*acta1b*	Actin, alpha 1b, skeletal muscle	Provides instructions for making the skeletal muscle α‐Actin, important for cell movement and muscle contraction; embryonic heart tube development	Skeletal muscle tissue: heart, ocular muscles; hypaxial muscle; developing pectoral fins	Possibly detectable in feces or urine
*b2m*	Beta‐2‐microglobulin	Associated with proteins of histocompatibility complex; modulator of lymphocyte surface and immune system regulator; involved in the presentation of peptide antigens to the immune system	Expressed in most nucleated cells, including immune cells	Possibly detectable in feces or urine
*rpl13a*	Ribosomal protein L13a	Promotes rRNA folding to form functional 3D structures during rRNA processing and stabilizes final spatial conformation of ribosome; component of the 60S subunit of the ribosome; component of the IFN‐gamma‐activated inhibitor of translation complex for inflammatory gene repression	Cytoplasm; higher levels in ovary and pancreatic tissues	Likely detectable in feces and female reproductive cells

### 2.7 RT‐qPCR Analysis

Each sample (eRNA and tissue) was investigated by RT‐qPCR with a suite of 10 genes (Table 2) using custom QIAGEN *Danio rerio* RT^2^qPCR Primer Assays—seven functional (PPAR pathway) and three reference gene assays. Zebrafish were the only fish involved in the experiment (and the only fish present in the lab where the experiment took place), ensuring minimal risk of off‐target amplifications. A total of four biological replicate samples per treatment (control and PFOS‐exposed) and two no template control samples (NTCs; negative control to confirm RT‐qPCR runs are contamination‐free), all including three RT‐qPCR technical replicates, were run for each combination of gene (*n* = 10) and timepoint (*n* = 9). Positive controls (RNA extracted from zebrafish tissue) were run separately to minimize the risk of contamination. All RT‐qPCR reactions were run in 25 μL volumes with 12.5 μL RT^2^SYBR Green Mastermix (QIAGEN), 1 μL of 10 μM RT^2^ qPCR primer assay (QIAGEN), and 1 μL of cDNA template. cDNA templates were not normalized before RT‐qPCR because one of the study goals was to determine at which timepoint amplification ceased (and thus gene detection ceased). The thermal cycler program was the same for all genes (both functional and reference) and included an initial denaturation step at 95°C for 10 min, followed by 40 cycles of 95°C for 15 s and 60°C for 1 min (Applied Biosystems ViiA 7 Real‐Time PCR System).

Careful quality assurance steps (Downey 2015) were completed for the entire RT‐qPCR dataset to eliminate false positive detections. These steps included an initial review of outlier cycle threshold (CT) values (those observed for eRNA samples that were below those observed for tissue samples) and imperfect melting temperature (Tm) values (more than one Tm or a single Tm at the lower limit of 60°C–62°C). Each flagged replicate was then visualized both on the amplification plot and melt curve plot to determine whether the replicate matched visualizations of other replicates for that gene and timepoint combination. Specifically, amplifications remained in the dataset if a single Tm was noted and that Tm matched those of other amplified samples in the same run of the same assay, the melt curve plot displayed a single peak, and the amplification plot showed three distinct phases (a baseline, an exponential phase, and a plateau), or if at least one Tm (if more than one) matched those of other amplified replicates in the same timepoint of the same assay, the melt curve plot displayed only one or two clean peaks, and the amplification plot showed three distinct phases (a baseline, an exponential phase, and a plateau). Assays remained in the final dataset if (1) sample amplifications were verified as noted above, (2) at least two biological replicates per treatment per assay had verified amplifications, and (3) there were zero amplifications in NTCs for that assay.

Once the RT‐qPCR dataset was quality assured, we examined the expression of the three reference gene transcripts to determine the frequency of detection as well as the variance in signal for each treatment (control and PFOS‐exposed) in both the tissue and all e‐mRNA timepoints. Among the three reference genes, rpl13a had the lowest variance for both the tissue and e‐mRNA samples, while also having the highest frequency of positive detections in the e‐mRNA samples. Therefore, we utilized rpl13a to conduct relative quantification analysis for the remaining gene set (seven function genes and two reference genes) using the ΔΔCT method (Schmittgen and Livak 2008). Once the ΔΔCT‐based expression values were calculated, the expression for all gene transcripts was standardized by the rpl13a expression level, allowing quantitative comparison between the control and PFOS exposure treatments for each gene target as well as a relative expression comparison among the full gene set.

### 2.8 Statistical Analyses

The RT‐qPCR datasets standardized to rpl13a expression were analyzed using a two‐way analysis of variance (ANOVA) individually for fish tissue and each e‐mRNA timepoint. The two‐way ANOVA (*p* = 0.05) tested for differences in transcriptional expression among genes in addition to the effect of PFOS exposure on expression for each gene relative to the control group. To assess if each dataset met the assumptions of this parametric statistical analysis, the Shapiro–Wilk test was used to evaluate the data for normality (*p* = 0.05), and homogeneous variance was assessed using the Brown‐Forsythe test (*p* = 0.05). The untransformed data did not meet the assumptions of ANOVA; therefore, the data were rank transformed, which enabled the datasets to conform to the assumptions of the parametric ANOVA procedure. The Holm‐Sidak method was used for pairwise tests of transcriptional expression comparing the treatment groups, as well as transcriptional expression relative to the designated reference gene, rpl13a. All statistical analyses were conducted using SigmaPlot/SigmaStat v. 13.0 software (Systat Inc. Palo Alto, CA).

## 3 Results

### 3.1 Analytical Chemistry for PFOS and PFOS Effects Assessment

Chemistry analyses of water samples exhibited expected PFOS levels in both control (all timepoints < 0.07 μg/L PFOS) and PFOS exposure (mean of 84.5 μg/L PFOS across timepoints) tanks (Table 3). Tissue samples (*n* = 4) also exhibited expected, bioaccumulated PFOS levels in both control (40 ± 20 μg/kg PFOS) and PFOS‐exposed (37,200 ± 16,700 μg/kg PFOS) fish. While both weight (*t* (190) = 1.355, *p* = 0.177) and length (*t* (190) = 1.674, *p* = 0.096) trended downward in PFOS‐exposed fish, neither was significantly decreased at *p* ≤ 0.05.

**TABLE 3:** Analytical values for PFOS in water samples collected from the 0 μg/L (control) and 100 μg/L PFOS exposures (*n* = 4 per treatment over exposure days 0–21) as well as in whole zebrafish tissue samples (*n* = 8 per treatment, including one male and one female from each tank) collected at the completion of the 21‐day exposure.

Exposure	Water chemistry collection day	Water PFOS concentration (μg/L)	Mean tissue PFOS concentration (μg/kg)
Control	0	< LOD	40 ± 20^a^
	1	0.04	
	11	0.04	
	21	0.07	
PFOS‐exposed	0	105	37,200 ± 17,000
	1	87	
	11	85	
	21	57	

Abbreviation: LOD, limit of detection.

^a^ Fish reared in holding tanks may have been exposed minimally to water and/or food containing PFOS.

### 3.2 Effect of Time on eRNA Abundance and e‐mRNA Gene Transcript Detection

**FIGURE 1:**
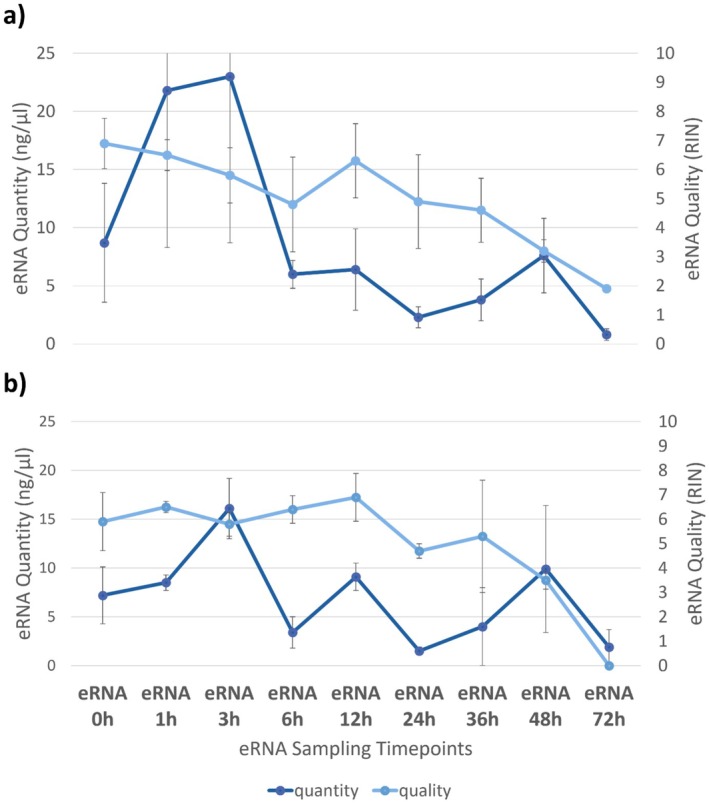
Metrics for the quantity and quality of total eRNA (not species‐ or gene‐specific) from adult *Danio rerio* exposed to PFOS at (a) 0 μg/L (control) or (b) 100 μg/L (PFOS‐exposed) for 21 days. Plotted values for quantity and quality represent means and standard deviations for eRNA samples collected at nine timepoints: ERNA 0 h (fish present) and eRNA 1–72 h (1–72 h after fish were removed from exposure tanks).

**FIGURE 2:**
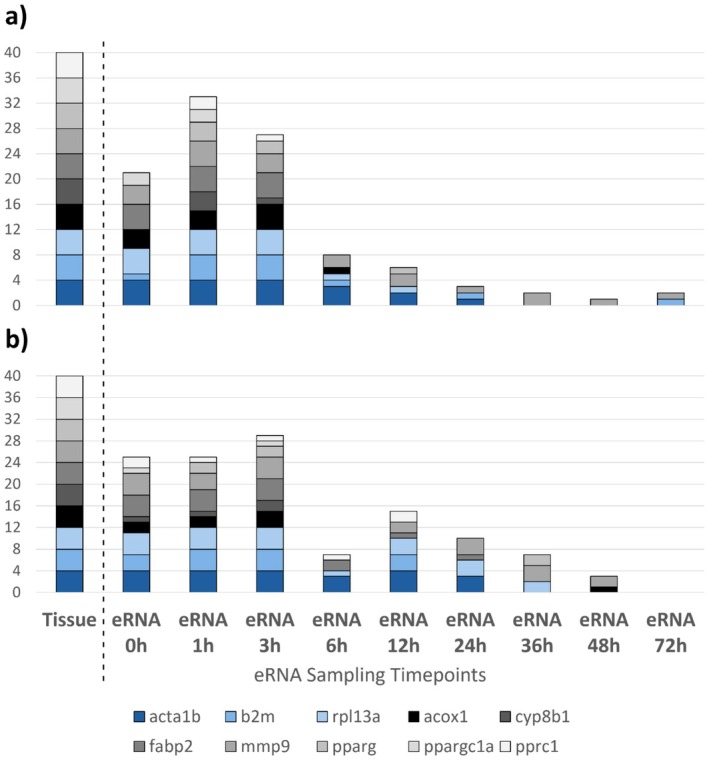
Number of gene transcript detections by biological replicate (*n* = 4 per treatment) of *Danio rerio* tissue mRNA, assayed as a quality control measure to confirm reverse transcriptase quantitative polymerase chain reaction (RT‐qPCR) success, and e‐mRNA samples collected at nine timepoints: ERNA 0 h (fish present) and eRNA 1 h–72 h (1–72 h after fish were removed from exposure tanks). Bar plots represent positive detections of 10 e‐mRNA gene transcripts in tanks of adult *D. rerio* (*n* = 24 per 8 L tank) exposed to PFOS at (a) 0 μg/L (control) or (b) 100 μg/L (PFOS‐exposed) for 21 days. The blue bars represent three reference genes (acta1b, b2m, rpl13a) and the black‐and‐gray bars represent seven genes involved in the peroxisome proliferator‐activated receptor (PPAR) signaling pathway (acox1, cyp8b1, fabp2, mmp9, pparg, ppargc1a, pprc1).

Total eRNA quantity and quality did not vary significantly by treatment, but decreased by timepoint (Figure 1). Tissue positive control samples had mean RNA quantities of 32.9 ± 8.1 ng/μL (control) and 25.8 ± 4.2 ng/μL (PFOS‐exposed), with associated Nanodrop 260/280 ratios of 1.80. Positive detection of all 10 e‐mRNA targets (seven functional and three reference genes) was observed for all four positive control tissue samples, including all technical replicate qPCRs (Figure 2). For e‐mRNA, positive detections were evident across all samples and genes up to 3 h post fish removal (Figure 2). After 3 h, detection rates declined steadily, which was mirrored by an overall decline in total eRNA concentrations (Figure 1); RIN declined as well, but the decrease appears to have commenced about 12 h post fish removal, with a pronounced decline 36 h post fish removal. Of the PPAR‐related target genes, acox1, fabp2, and mmp9 e‐mRNAs were detectable for the longest amount of time in water samples collected post fish removal. Investigation of transcriptional expression in the zebrafish tissue samples indicated significant expression differences for all PPAR‐related genes relative to the reference genes; however, no significant differences in transcriptional expression were observed between the treatment groups (control vs. PFOS‐exposed). Likewise, investigation of e‐mRNA expression did not identify significant differences in expression relative to the reference genes between treatment groups (control vs. PFOS‐exposed).

## 4 Discussion

### 4.1 Observation on e‐mRNA Decay

We attempted to observe whether exposure to an environmentally relevant concentration of PFOS (100 μg/L) would result in detectable shifts in the concentrations of e‐mRNAs for genes known to be sensitive to PFOS exposure in zebrafish, but found no evidence of such an effect. However, we did detect many of those e‐mRNA targets and were able to track their concentrations over time indirectly through changes in the frequency of samples with amplified detections for those markers. While we noted apparent counterintuitive increases in total eRNA quantity from one time point to a subsequent time point, this phenomenon is reported in other similarly structured studies of eDNA and eRNA decay (Xu and Asakawa 2025; Mugunthan et al. 2023; Lance and Guan 2020; Eichmiller et al. 2016). The apparent increases may simply be a function of stochasticity in the RNA concentrations procured in different e‐mRNA samples, among subaliquots injected into qPCR solutions, and/or in amplification dynamics for target‐sparse qPCR, as well as potential impacts from adsorbed nucleic acids desorbing from biofilms and other surfaces (Eichmiller et al. 2016; Mugunthan et al. 2023). A strong decline in both total eRNA concentrations and sample‐level detection rates across e‐mRNA gene targets occurred between Hour 3 and Hour 6 post fish removal, continuing steadily to nearly complete extinction of signal from the focal e‐mRNAs (Figure 1). The drop in detection for timepoints beyond 3 h would be less important if monitoring a system where the target organism is still present and the goal is to detect stressor effects (e.g., changes in gene expression) as they are occurring (e.g., early detection of an environmental impact); however, the loss in detections beyond 3 h for e‐mRNA gene targets could be beneficial to establishing a timeline of presence for target organisms or target gene responses.

An interesting pattern observed in our study was that total eRNA quality, as characterized with the RIN score, did not exhibit a significant decline until at least 9 h after detections of e‐mRNAs had undergone significant decline (which corresponded to the drop in total eRNA quantity that occurred after the eRNA 3 h timepoint). RIN computation is complex and based on numerous features of the RNA pool in a sample (Schroeder et al. 2006), and is substantially influenced by the decay of large ribosomal RNAs (rRNAs; e.g., 18S, 28S). The pattern we observed indicates that e‐mRNA degrades more rapidly than e‐rRNA (environmental rRNA). We also note that the total eRNA detected in our study appeared to be of only moderate quality, even at the start of sampling (mean of 6.5 on the RIN scale of 1–10, with 10 = highest RNA integrity). This is not surprising given the source (i.e., dead cellular and genetic material shed into the environment) of eRNA.

An important factor in future applications of e‐mRNA as a source of biological evidence for stressor presence and impact is consideration of whether the organs or tissues in which stressor‐linked gene expression changes are expected to occur are also likely sources for eRNA (Hiki and Jo 2025; Stevens and Parsley 2023). Similar to eDNA, deposited eRNA is expected to be derived largely from excretory tracts and materials (e.g., feces, urine), broadcast gametes, and cells associated with external epithelia (e.g., gills, slime coating; Hiki and Jo 2025). In this study, PFOS bioconcentrated in tissues (Table 3) to similar levels previously observed in long‐term aquatic exposures in zebrafish (Gust et al. 2025). Though liver, kidney, gut, gonad, and brain cells are known to exhibit gene expression shifts in PFOS‐exposed organisms, we did not know a priori to what degree our selected gene‐transcript targets associated with the PPAR pathway would be expressed in and deposited into the environment from other organs and tissues. Our selection of gene targets to track patterns of e‐mRNA degradation was instead strongly influenced by interest in the effects of PFOS (at environmentally relevant concentrations), a high priority class of contaminant, and the genes known to be sensitive to this stressor. In initial trials with non‐experimental samples to select PFAS‐related genes for use with eRNA samples (see Section 2.6), all 13 tested genes were successfully amplified in tissue samples while only seven amplified in eRNA samples (Table 1), demonstrating that not all mRNAs expressed in tissues will translate to expression in e‐mRNA. A potential solution to data gaps as to which genes may provide the most reliable e‐mRNA data for potential stressor presence and impacts is to conduct broad scans of e‐mRNAs in water samples, under stronger treatment conditions, using advanced molecular genetic tools such as shotgun RNA sequencing (RNAseq).

### 4.2 How to Treat e‐mRNA Data

Though our study did not, unfortunately, result in any observable differences in e‐mRNA detection levels between treatments (PFOS‐exposed vs. control) and thus little need for in‐depth analysis, the question of how to treat e‐mRNA data is intriguing. Conventionally, toxicogenomic studies of stressor‐associated gene regulation focus on fold changes determined from standardized differences in mRNA copy numbers among control and treatment groups. The mRNA copy numbers are extrapolated from the CT values obtained during RT‐qPCR. Fold changes are standardized against observed fold changes in a reference gene that is expected to show consistent, conserved expression regardless of treatment (stressor‐exposed or non‐treatment). The endpoint or metric for these gene expression trials is ΔΔ_CT_ or 2^ΔCT^ or, somewhat simply, the change in the copy number of the gene of interest in the treatment group relative to the gene copy number in the control group (Δ_I_), all relative to or standardized by the difference between reference gene copy numbers (Δ_H_) in the same treatment and control groups (2^ΔCT^ = Δ_I_/Δ_H_). However, in the field of eDNA, the majority of studies rely on simple binary outcomes of detection vs. non‐detection, eschewing more quantitative CT or estimated DNA target copy number data. This is, in part, because eDNA often occurs at levels very near the limits of detection and usually below the limits of quantification (Guri et al. 2024; Lesperance et al. 2021). Another important factor is that the relationship between estimated DNA copy numbers and the quantity of source material in the system is often weak or complex (as inferred from the complicated, often poor quantitative associations between eDNA and organism abundance or biomass; Rourke et al. 2022). Simple detection/non‐detection results have been viewed as more reliable and actionable. In the case of e‐mRNA, it is likewise possible that there will generally be weak or no correlation between estimated e‐mRNA copy numbers and fold changes in organismal tissues and cells—though this phenomenon has yet to be explored, demonstrated, or reported in the published literature. Instead, like most eDNA and eRNA studies to date, the application of e‐mRNA (or other RNAs whose abundance may reflect organismal physiological states) will likely require the development of an alternative assessment that makes use of detection/non‐detection outcomes.

In one potential approach, a simple threshold of comparative detection (positive qPCR amplification) frequencies for a reference gene and a target gene might be reliably informative. For example, in a set of water samples taken under non‐stressed (control) conditions, 90%–100% detections of one or more reference genes and 70%–100% detection of the target gene might be anticipated, while under stressor exposure, 90%–100% detections of reference genes and 0%–50% detection of the target gene might be indicative of down‐regulation of that gene. Detection of the target gene at a frequency of 51%–69% might be considered a region of inferential uncertainty. Expanding this approach to criteria based on the ratio of detection frequencies, for example, a 2:1 detection frequency ratio for reference gene: target gene (or multiple ratios for multiple reference genes) could be seen as essentially equivalent to the ΔΔ_CT_ for copy number data. An added benefit of incorporating reliably amplified reference genes into the approach is that thresholds for minimum reference gene detection rates might serve as criteria for whether or not a water sample may have sufficient total eRNA integrity and concentrations for robust testing. We also note that the assessment of detection frequencies for multiple reference genes roughly mirrors the recommended use of multiple assays per taxon of interest in eDNA surveys. The multiple assay approach provides a “hedge” against random sampling error in capturing detectable numbers of intact strands of sparse, degraded target DNA when sampling water, aliquoting DNA extracts for PCR, etc. In the case of e‐mRNA, employing multiple reference genes might provide a similar hedge. If there are multiple genes of interest related to physiological, cellular, or genetic responses to a stressor, or even multiple RT‐qPCR loci within a target gene, then a multiple assay approach would again be recommended.

A second potential approach would take advantage of technical replicates, or multiple RT‐qPCRs for the same assay run with cDNA templates from the same RNA extraction from the same eRNA sample. Typical eDNA and eRNA surveys incorporate multiple technical replicates per assay per sample (e.g., 3–8 qPCRs or RT‐qPCRs). A semi‐quantitative approach would be to calculate an index‐based ratio of the mean or median number of technical replicates that are positive for the e‐mRNA target to the mean or median number of technical replicates that are positive for one or more reference genes. Comparison of index value between sites might then provide an indication of whether or not suspected stressors were in play. Such an approach might suffer substantially from random sampling error when the number of technical replicates is small, and additional interesting questions might revolve around the statistical associations between the number of technical replicates and inferential reliability.

This study demonstrates the importance of target gene selection when using e‐mRNA to screen for indications of stressor‐specific transcriptional changes. While we were able to isolate and show amplification of target e‐mRNAs, we did not see any significant differences between control and PFOS‐exposed eRNA samples. Care must be taken, as even genes which are differentially expressed in response to a target stressor in animal tissue studies may not be deposited in large quantities in an environmental matrix, especially if the gene assays target organ(s) that do not readily transport mRNA products into the environment. In such cases, preliminary studies conducted using RNAseq of collected eRNA would allow for identification of target e‐mRNAs that are both reliably deposited in the environmental matrix and indicative of stressor exposure (e.g., PFOS). As with any emerging and rapidly growing methodology, best practices for eRNA and e‐mRNA detection are evolving with each new study published, and the present study contributes observations, results, lessons learned, and recommendations for the continued advancement of e‐mRNA methods for consideration by the community of practice.

## Author Contributions

**Denise L. Lindsay:** conceptualization (equal). **J. Erik Mylroie:** conceptualization (equal). **Kurt A. Gust:** conceptualization (equal). **Elijah M. Cowan:** conceptualization (equal). **Richard F. Lance:** conceptualization (equal).

## Conflicts of Interest

The authors declare no conflicts of interest.

## Data Availability

Raw data underlying the main results of the study is archived in the US Army Engineer Research and Development Center (ERDC) Environmental Laboratory Knowledge Core (https://doi.org/10.21079/11681/49841).
